# Predictors of employment attrition in Lebanon during multifaceted crises: The role of chronic diseases – a national cross-sectional study

**DOI:** 10.1371/journal.pone.0328028

**Published:** 2026-03-25

**Authors:** Myriam Dagher, Ali Abboud, Ghada E. Saad, Rita Itani, Rindala Fayyad, WOMENA Study Group, Hala Ghattas, Stephen J. McCall

**Affiliations:** 1 Center for Research on Population and Health, Faculty of Health Sciences, American University of Beirut, Beirut, Lebanon; 2 Department of Economics, Faculty of Arts and Sciences, American University of Beirut, Beirut, Lebanon; 3 Department of Health Promotion, Education, and Behavior, Arnold School of Public Health, University of South Carolina, Columbia, South Carolina, United States of America; The University of Warwick, UNITED KINGDOM OF GREAT BRITAIN AND NORTHERN IRELAND

## Abstract

The COVID-19 pandemic and Lebanon’s ongoing economic crisis exacerbated existing workforce and health disparities. This study explored the predictors of employment attrition during Lebanon’s concurrent crises and examined the association between chronic conditions and employment attrition. This cross-sectional study recruited adults aged 19–64 years residing in Lebanon through random digit dialing (January – July 2024). Data collected included socio-demographics, household characteristics, employment history and characteristics, and self-reported chronic conditions. The outcome was loss of paid employment (employment attrition) during the crises. Predictors were identified through a Least Absolute Shrinkage and Selection Operator (LASSO) regression, and model discrimination and calibration were assessed. Logistic regression models, adjusted for covariates identified through a directed acyclic graph, estimated the associations between the presence and types of chronic conditions and employment attrition. The effect modification by age on the association between chronic conditions and employment attrition was also assessed. Of 2103 participants employed prior to the onset of the concurrent crises (pre-2020), 72.7% were males, 70.1% were Lebanese, and 14.7% became unemployed during the crises. Predictors of employment attrition were: older age, female sex, non-Lebanese nationality, being married, having no formal education, having at least one of either CVD, diabetes, or musculoskeletal disorders, working in a private business or non-governmental institution, and having an oral agreement with employer. The prediction model had a moderate discriminative ability and good calibration. Pre-existing cardiovascular disease (adjusted odds ratio (aOR): 2.15; 95% confidence intervals (CI), 1.27 to 3.64) and diabetes (aOR: 2.52; 95% CI, 1.43 to 4.45) were independently associated with employment attrition. The ORs of employment attrition comparing those with to those without musculoskeletal disorders significantly increased with age. This study underscores the importance to address life-course disparities that contribute to employment attrition and to consider proactive job protections to mitigate workforce disruptions during times of crises, particularly in contexts where social safety nets are absent.

## Introduction

Decent and inclusive work for all, indicated in the United Nations Sustainable Development Goal 8, is essential for sustainable economic growth and better health outcomes [1]. Despite the reduction in the global unemployment rate to around 5%, inequalities in the labor market still persist [2]. These disparities are influenced, and often shaped, by factors rooted in the individual’s life course. Theoretical frameworks, such as life-course approaches, highlight how early life experiences and accumulated risks across one’s life span shape employment stability and work trajectories [3]. Different life exposures at different time points can have direct or indirect impacts on the likelihood of unemployment, precarious employment, or early retirement [4].

Macro-level environmental factors, such as the benefit system and economic development, and meso-level factors, like workplace characteristics and working conditions, can affect the capability of working-age individuals to remain in the workforce [5]. For instance, workers in physically demanding occupations, such as in construction, experienced shorter working lives by about a year and exited the labor force prematurely [6–8]. Additionally, poor compensation and temporary contracts were found to predict employee attrition and employment turnover [9,10]. Micro-level factors such as sex, age, and education also influence transitions into and out of the labor force. For example, people with primary or secondary education spent more years unemployed than those with tertiary education [11]. Chronic health conditions are also important micro-level barriers to employment, especially conditions that limit functionality at work, such as cardiovascular disease, diabetes, and musculoskeletal disorders [5,12,13]. These life course factors act as transitions that alter a person’s employment trajectory. They are particularly impactful when they occur during sensitive periods, such as when individuals are combining career demands with parenthood and caregiving responsibilities. Moreover, macro-, meso-, and micro- level factors can accumulate over time and may interact across the life course to increase the likelihood of employment transitions [4].

Lebanon, a low-middle-income country (LMIC) in the Middle East and North Africa (MENA) region, is characterized by low labor force participation, especially among women [1]. Since 2019, Lebanon has been grappling with concurrent crises: from a protracted political and economic crisis to the COVID-19 pandemic and the Beirut Port explosion [14,15]. The labor underutilization rate in the country dramatically increased from around 16% between 2018 and 2019 to 50% in 2022, accompanied by a gender gap in employment [1]. The concurrent crises have added another layer of complexity to the already existing health challenges and workforce disparities in the country [1,16].

Prior studies examining determinants or factors of unemployment or workforce transitions have primarily focused on high-income settings. This leaves knowledge gaps in understanding the drivers of employment attrition in LMIC populations, including Lebanon [17]. Studies exploring factors associated with employment transition in LMIC settings, particularly in the context of overlapping crises, are scarce. For instance, while one recent study across five MENA countries explored changes in employment during the pandemic, it focused solely on women with care responsibilities [18]. In addition, labor studies in Lebanon generate estimates of labor market indicators without examining their links to chronic health conditions [1]. This makes it particularly important to understand the factors that may have forced transitions from paid employment to unemployment during turbulent periods. Therefore, this study explored the predictors of employment attrition during Lebanon’s concurrent crises and examined the association between pre-existing chronic conditions and employment attrition.

## Materials and methods

### Study design and setting

This was a national cross-sectional study conducted as part of a larger research project entitled “Identifying opportunities to improve the lived experience and health of working women in the MENA: from COVID to recovery” (WOMENA). The main aim of the project was to examine trends in the gender distribution of labor force participation in the MENA region and how these trends impacted health and well-being, with a particular focus on the impact of the COVID-19 pandemic. This study represented a component of the larger research project and specifically aimed to understand the intersection between labor force participation and health in Lebanon. The study protocol was reviewed and approved by the American University of Beirut, Social and Behavioral Sciences Institutional Review Board (IRB) [Reference: SBS-2023–0182]. Oral consent was obtained from all participants prior to their involvement in the study. Verbal consent was recorded, and a soft copy of the consent form was shared with the participants. This consent procedure was approved by the IRB. All necessary measures have been taken to protect participants’ privacy.

### Sampling and study population

Participants were recruited through random digit dialing (RDD) between January and July 2024. Random phone numbers were generated using the 11-digit structure for mobile phone numbers in Lebanon. These numbers had the first three digits corresponding to the international country calling code for Lebanon (961), followed by two digits indicating the country’s mobile network operators (03, 70, 71, 76, 78, 79, or 81), and the remaining six digits were randomly generated. Each number generated was dialed with a maximum of two call attempts. If respondents were unable to answer or complete the survey at the time of the initial call, follow-up call appointments were scheduled.

Data were collected by trained data collectors in the participants’ mother tongues and data entry was performed using SurveyCTO software [19]. A comprehensive data monitoring plan and standardized data quality procedures were implemented during data collection on a weekly basis to identify and address systematic errors in real-time. As part of the process, 5% of surveys were recorded and cross-checked against the actual data entered, which gave an average error rate of 0.6%.

Respondents completed a set of eligibility screening questions. The survey was restricted to working age adults (aged 19–64 years) who were permanently residing in Lebanon at the time of the survey. Respondents who were on a visit or temporary stay were not eligible to participate in the survey. There were no restrictions based on citizenship or legality of residency status. Employed women were oversampled given the low female labor force participation in the population.

### Measures

#### Outcome measures.

The outcome of interest in this study was loss of paid employment (employment attrition) during the peak period of the concurrent pandemic and economic crisis in Lebanon (2020–2023) [14,15]. The survey included an employment module with questions on current and retrospective employment. Participants were asked about the start and end dates of every job they had in the past 10 years. The data allowed the construction of the outcome, employment attrition, a binary variable that assigned the value of 1 if the individual “left employment during the crises” (between 2020 and 2023), and 0 if the individual “remained employed after the crises”. The sample was restricted to those who were employed prior to the onset of the crises (n = 2103). Additionally, participants who left employment were asked about the reasons for their work discontinuation.

#### Candidate predictors and data.

Ten candidate predictors for employment attrition were identified through the literature and included in the model development. These predictors were age, sex, nationality, marital status, education, urbanization of living environment, number of children, job sector prior to the crises, contractual agreement prior to the crises, and presence of pre-existing (prior to the crises) chronic conditions, including cardiovascular disease (CVD), diabetes, or musculoskeletal disorders. These chronic conditions were selected as they have been shown to be associated with early retirement or reduced labor market participation [20–22].

The urbanization of living environment was assessed through a single-item self-report measure “Please indicate how urban your living environment is on a 7-point scale from 1 (not urban at all) to 7 (very urban).” The responses were then presented as binary, with a score of 6 or more indicating *extremely urbanized* areas [23]. This measure showed strong correlation with objective measures of urbanization [23]. Self-reported chronic conditions were measured by asking participants if they had ever been diagnosed or informed by a healthcare professional about any of the following chronic conditions or diseases: CVD, diabetes, musculoskeletal disorders, hypertension, chronic respiratory disease, chronic kidney disease, and neurodegenerative conditions. Participants’ recall of their date of diagnosis enabled the distinction between pre-existing and current chronic conditions.

### Statistical analysis

Absolute frequencies and weighted proportions were presented for categorical variables, and medians with their interquartile range (IQR) for continuous variables. Weighted odds ratios (ORs) alongside their 95% confidence intervals (CIs) were calculated using unadjusted logistic regression models to examine the odds of employment attrition for each independent variable. All candidate predictors were categorical except age and number of children, which had a linear association with employment attrition. Coefficient estimates with p-values less than 0.05 were considered statistically significant. Sampling and post-calibration weights were calculated and applied to the analysis to allow for national estimates.

A Least Absolute Shrinkage and Selection Operator (LASSO) logistic regression model was used to identify the predictors of employment attrition during the concurrent crises for the study sample. All candidate predictors were entered for model development, where the penalty strength (λ) was selected using 10-fold cross-validation (S1 Appendix). A logistic regression was run to produce adjusted ORs and 95% CIs of selected predictors.

The model discrimination was assessed through C-Statistic/Area Under the Receiver Operating Characteristic Curve (AUC), ranging from 0.5 to 1 with an index of 1 indicating perfect discrimination ability between participants who experienced employment attrition from those who did not  [24]. The evaluation of the model calibration (agreement between predicted and observed risk) was assessed through a calibration plot, calibration slope (C-Slope), and calibration-in-the-large (CITL). A perfectly calibrated model is illustrated by a diagonal line with an intercept of 0 and a slope of 1 [25]. No variable had more than 2.0% missing data. Missing data was assessed using the Little’s test and was found to be missing completely at random. A complete case analysis was performed [26]. All analyses were conducted using Stata/SE statistical software version 18 (StataCorp LLC) and R version 4.4.2.

#### Association between pre-existing chronic conditions and employment attrition.

A secondary analysis explored the association between the presence and types of pre-existing chronic conditions (exposures) and employment attrition during the crises (outcome). The outcome employment attrition was analyzed as a binary variable. The presence of pre-existing chronic conditions, reported to be diagnosed prior to the onset of the concurrent crises, was categorized into two binary variables: i) at least one chronic condition from all listed chronic conditions, and ii) at least one chronic condition known to be associated with early retirement or reduced labor market participation (CVD, diabetes, or musculoskeletal disorders).Types of pre-existing chronic conditions were also treated as binary variables, categorizing participants based on a self-reported pre-existing history of CVD, diabetes, musculoskeletal disorders, hypertension, chronic respiratory disease, chronic kidney disease, and neurodegenerative conditions.

A Directed Acyclic Graph (DAG) was constructed using DAGitty software (dagitty.net) to identify the minimum set of covariates to control for in the analysis [27–29]. It included key variables, as informed by the literature [17,30–33]. The initial DAG was validated by three members of the study team and underwent minor modifications based on consensus (S2 Fig).

The minimal sufficient adjustment set included age, sex, marital status, job sector before the crises, and job income before the crises (in USD per year, a continuous variable). Separate logistic regression models were used to assess the association between the presence and types of chronic conditions and employment attrition after adjusting for the identified covariates. Weighted unadjusted and adjusted ORs were calculated with their associated 95% CIs. To assess whether the associations between chronic conditions and employment attrition varied by age, we tested for interactions between the presence and types of chronic conditions and age. For significant interactions (p-value < 0.05), we estimated the ORs of employment attrition comparing those with to those without the exposure at selected ages between 19 and 64 years. The odds of employment attrition at different ages were plotted for individuals with and without the exposure to illustrate differences in the likelihood of losing work as age increases.

## Results

**Fig 1:**
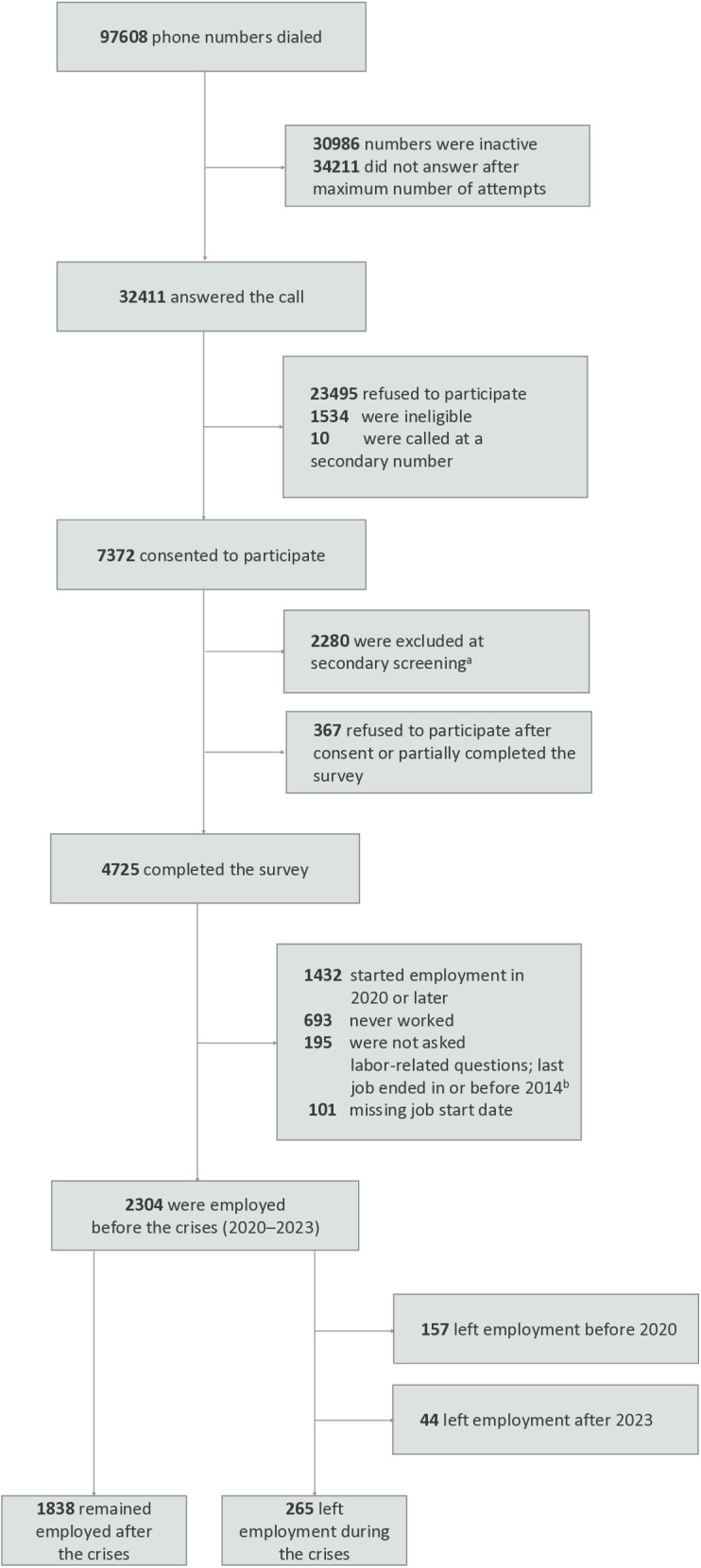
Flow diagram of participant selection of adults employed in Lebanon prior to the concurrent crises. ^a^ At this stage, we further assessed participants’ employment status and sex to enable oversampling of employed women in line with national estimates. ^b^ When collecting retrospective data on employment, we did not ask about employment characteristics and history for individuals whose last job fell beyond 10-year recall period (i.e., whose last job ended in or before 2014).

A total of 97608 phone numbers were contacted through RDD, of whom 7372 consented to participate. Among them, 4725 completed the full survey. After exclusions, a total of 2304 participants were employed before the crises (2020–2023). The final study sample for analysis comprised 2103 participants who either remained employed after the crises (n = 1838) or left employment during the crises (n = 265) (Fig 1).

The characteristics of the study sample are reported in S1 Table. The median age of the participants in the study sample was 40 years (IQR: 33–49 years); 1466 participants (72.7%) were males and 1311 (70.1%) were Lebanese. Of the study sample, 265 (14.7%) participants left employment during the crises, while 1838 (85.3%) remained employed after the crises (S1 Table). The reasons reported for leaving work during the crises included illness, disability, or injury (23.0%); ending economic activity through resignation or retirement (18.1%); temporary or seasonal job (17.7%); inappropriate working conditions related to working hours, salary, work environment, or logistical challenges accessing the workplace (17.1%); family sickness or responsibilities, such as family illness, caregiving, marriage, or pregnancy (16.8%); and dismissal or redundancy (11.1%) (S1 Fig). Of the study sample, 310 (15.4%) participants reported having at least one pre-existing chronic condition, including 167 (7.9%) who had musculoskeletal disorders, 125 (6.6%) CVD, and 94 (5.1%) diabetes (S1 Table).

The weighted unadjusted ORs and 95% CIs of the association between employment attrition during the crises and each study sample characteristic are reported in Table 1. Among the study sample, females were more likely to leave employment during the crises (OR, 2.61; 95% CI, 1.98 to 3.44), as were non-Lebanese (OR, 1.58; 95% CI, 1.21 to 2.05), those who had pre-existing CVD, diabetes, or musculoskeletal disorders (OR, 1.41; 95% CI, 1.00 to 2.00), those who worked in private businesses (OR, 2.45; 95% CI, 1.44 to 4.17) or in non-governmental institutions (OR, 3.81; 95% CI, 1.75 to 8.31) prior to the crises compared to those who worked in governmental institutions, and those who had an oral agreement with their employer prior to the crises (OR, 2.20; 95% CI, 1.51 to 3.20) compared to those who had written contracts.

**Table 1:** Characteristics of the study sample stratified by employment attrition during the concurrent crises in Lebanon (2020-2023) (n = 2103).

	Participants, No. (Weighted %)^a^			
	Remained employed after the crises (n = 1838)	Left employment during the crises (n = 265)	Weighted Unadjusted OR (95% CI)^b^	p-value
Age, median (IQR), years	40 (33-49)	40 (32-51)	1.00 (0.99 to 1.16)	.676
Sex				
Male	1293 (88.9)	173 (11.1)	1 [Reference]	
Female	545 (75.5)	92 (24.5)	2.61 (1.98 to 3.44)	<.001
Nationality				
Lebanese	1178 (87.1)	133 (12.9)	1 [Reference]	
Non-Lebanese	660 (81.0)	132 (19.0)	1.58 (1.21 to 2.05)	.001
Marital status				
Married	1379 (84.9)	209 (15.1)	1 [Reference]	
Non-married^c^	459 (86.3)	56 (13.7)	0.89 (0.65 to 1.24)	.499
Education				
Never attended school	48 (65.4)	19 (34.6)	1 [Reference]	
Formal or technical education	1296 (84.6)	204 (15.4)	0.34 (0.19 to 0.60)	<.001
Higher education	494 (89.1)	42 (10.9)	0.23 (0.12 to 0.44)	<.001
Urbanization of living environment				
Not urbanized/strongly urbanized	1420 (84.5)	222 (15.5)	1 [Reference]	
Extremely urbanized	398 (88.1)	41 (11.9)	0.74 (0.51 to 1.06)	.102
Missing	20	2	NA	NA
Children, median (IQR), No.	2 (1-4)	2 (1-4)	1.04 (0.98 to 1.10)	.249
Pre-existing^d^ chronic conditions linked with unemployment, No.^e^				
0	1571 (85.9)	215 (14.1)	1 [Reference]	
≥ 1	260 (81.2)	50 (18.8)	1.41 (1.00 to 2.00)	.053
Missing	7	0	NA	NA
Cardiovascular disease, pre-existing				
No	1723 (85.8)	238 (14.2)	1 [Reference]	
Yes	98 (75.6)	27 (24.4)	1.95 (1.23 to 3.09)	.004
Missing	17	0	NA	NA
Diabetes, pre-existing				
No	1752 (85.7)	243 (14.3)	1 [Reference]	
Yes	73 (75.7)	21 (24.3)	1.93 (1.15 to 3.24)	.013
Missing	13	1	NA	NA
Musculoskeletal disorders, pre-existing				
No	1684 (85.4)	240 (14.6)	1 [Reference]	
Yes	142 (82.2)	25 (17.8)	1.27 (0.79 to 2.03)	.319
Missing	12	0	NA	NA
Job sector, pre-2020^d^				
Government	254 (92.0)	17 (8.0)	1 [Reference]	
Private business	829 (82.5)	149 (17.5)	2.45 (1.44 to 4.17)	.001
Private household	56 (91.0)	4 (9.0)	1.14 (0.35 to 3.71)	.830
Non-governmental institution	61 (75.1)	14 (24.9)	3.81 (1.75 to 8.31)	.001
Freelance	635 (87.0)	80 (13.0)	1.72 (0.99 to 3.00)	.056
Missing	3	1	NA	NA
Contractual agreement, pre-2020				
Written	449 (89.4)	40 (10.6)	1 [Reference]	
Oral	756 (79.3)	165 (20.7)	2.20 (1.51 to 3.20)	<.001
Independent contractors^f^	595 (89.6)	56 (10.4)	0.97 (0.63 to 1.50)	.897
Missing	38	4	NA	NA

^a^Row percentages are used to show the distribution of the variables stratified by the outcome. Percentages are presented in parentheses. 95% Confidence Intervals (CIs) are shown in parentheses with “to” separating the range. The reference category is indicated using square brackets (e.g., 1 [Reference]). IQR refers to interquartile range. NA refers to not applicable.

^b^Dependent (outcome) variable is individuals who became unemployed at any time during the concurrent crises (2020–2023) compared to those who were still employed after the crises. OR: Odds Ratio; CI: Confidence Interval.

^c^Non-married includes participants who are single, engaged, divorced/separated, or widowed.

^d^Pre-existing or pre-2020 refers to conditions or circumstances that were present prior to the onset of the concurrent crises.

^e^At least one of the chronic conditions known to be associated with early retirement or reduced labor market participation: cardiovascular disease, diabetes, or musculoskeletal disorders.

^f^Independent contractors include owners, partners, and own account workers.

Additionally, participants living in extremely urbanized areas had lower odds of employment attrition compared to those living in less urbanized areas (OR, 0.74; 95% CI, 0.51 to 1.06). Those with formal or technical education (OR, 0.34; 95% CI, 0.19 to 0.60) or higher education (OR, 0.23; 95% CI, 0.12 to 0.44) were less likely to become unemployed during the crises than those who never attended school.

### Predictors of employment attrition and model performance

Table 2 presents the penalized coefficients, adjusted ORs, and their corresponding 95% CIs. Ten predictors of employment attrition were identified, including age, sex, nationality, marital status, education, urbanization of living environment, number of children, presence of pre-existing chronic conditions (CVD, diabetes, or musculoskeletal disorders), job sector prior to the crises, and contractual agreement prior to the crises. The coefficients indicated that older age, female sex, having non-Lebanese nationality, being married, never having attended school, having at least one pre-existing CVD, diabetes, or musculoskeletal disorders, working in a private business or non-governmental institution, and having an oral agreement with employer predicted employment attrition during the concurrent crises in Lebanon.

**Table 2:** Predictors of employment attrition during the concurrent crises in Lebanon.

Model Predictors	Codes	Penalized Coefficients^a^	Adjusted OR (95% CI)^b^
Age (years)		0.14	1.01 (1.00 to 1.03)
Sex			
Male	0	–	1 [Reference]
Female	1	0.57	1.79 (1.32 to 2.43)
Nationality			
Lebanese	0	–	1 [Reference]
Non-Lebanese	1	0.37	1.47 (1.06 to 2.05)
Marital status			
Married	0	0.21	1 [Reference]
Non-married^c^	1	–	0.80 (0.55 to 1.16)
Education			
Never attended school	0	0.77	1 [Reference]
Formal or technical education	1	–	0.46 (0.25 to 0.83)
Higher education	2	−0.54	0.26 (0.13 to 0.53)
Urbanization of living environment			
Not urbanized/strongly urbanized	0	–	1 [Reference]
Extremely urbanized	1	−0.58	0.55 (0.38 to 0.80)
Number of children		−0.04	0.95 (0.88 to 1.03)
Pre-existing^d^ chronic conditions linked with unemployment, No.^e^			
0	0	–	1 [Reference]
≥ 1	1	0.39	1.48 (1.02 to 2.15)
Job sector, pre-2020 ^d^			
Government	0	−0.29	1 [Reference]
Private business	1	0.21	1.68 (0.93 to 2.99)
Private household	2	−0.96	0.50 (0.15 to 1.98)
Non-governmental institution	3	0.48	2.20 (0.90 to 4.70)
Freelance	4	–	1.35 (0.70 to 2.63)
Contractual agreement, pre-2020			
Written	0	–	1 [Reference]
Oral	1	0.66	1.93 (1.23 to 3.04)
Independent contractors^f^	2	−0.11	0.88 (0.51 to 1.52)
Intercept		−3.16	0.08 (0.03 to 0.23)

^a^Penalized coefficients obtained using 10-fold cross-validation.

^b^Dependent (outcome) variable is individuals who became unemployed at any time during the concurrent crises (2020–2023) compared to those who were still employed after the crises. OR: Odds Ratio; CI: Confidence Interval.

^c^Non-married includes participants who are single, engaged, divorced/separated, or widowed.

^d^Pre-existing or pre-2020 refers to conditions or circumstances that were present prior to the onset of the concurrent crises.

^e^At least one of the chronic conditions known to be associated with early retirement or reduced labor market participation: cardiovascular disease, diabetes, or musculoskeletal disorders.

^f^Independent contractors include owners, partners, and own account workers.

**Fig 2:**
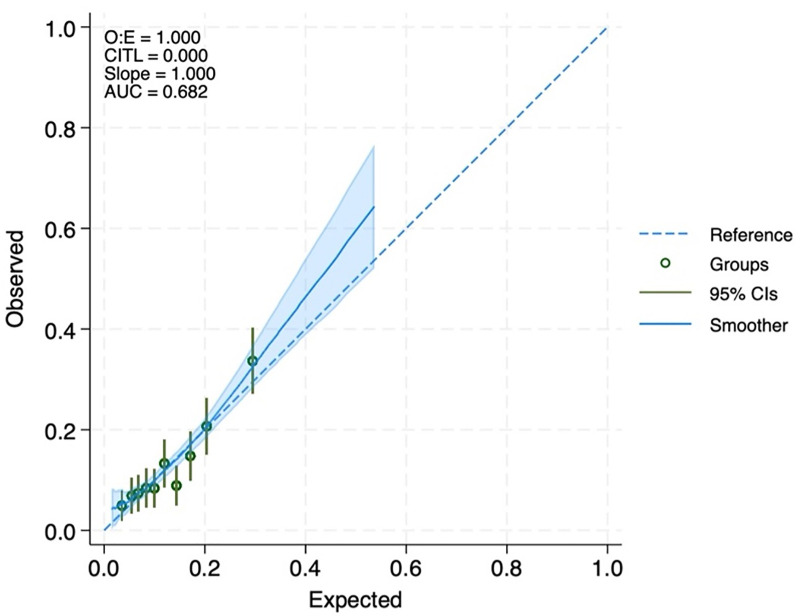
Calibration plot of the prediction model for employment attrition during the concurrent crises in Lebanon. The dashed diagonal line represents perfect calibration. Whiskers, 95% confidence intervals (CIs); O.E, observed-to-expected ratio; CITL, calibration-in-the-large; and AUC, the Area Under the Receiver Operating Characteristic Curve.

The model demonstrated a moderate discriminative ability with an AUC of 0.68 (95% CI, 0.65 to 0.72), reflecting its moderate ability to differentiate participants with and without employment attrition. The model also showed good calibration with a C-Slope of 1.00 (95% CI, 0.80 to 1.20) indicating that the model’s predicted risks align closely with observed outcomes (Fig 2).

### Chronic conditions and employment attrition

The weighted unadjusted and adjusted ORs for employment attrition by pre-existing chronic conditions are presented in Table 3. Participants with at least one of the following pre-existing chronic conditions – CVD, diabetes, or musculoskeletal disorders – had significantly higher odds of employment attrition during the crises (aOR: 1.57; 95% CI, 1.05 to 2.34), compared to those without any of these conditions. When considering each of these chronic conditions separately, participants with pre-existing CVD (aOR: 2.15; 95% CI, 1.27 to 3.64) or diabetes (aOR: 2.52; 95% CI, 1.43 to 4.45) had significantly higher odds of employment attrition compared to participants without these conditions.

**Table 3:** Association between employment attrition during the concurrent crises in Lebanon (2020-2023) and pre-existing chronic conditions.

	Weighted Unadjusted OR (95% CI)^a^	Weighted Adjusted OR (95% CI)^b^
Chronic conditions, No.^c^		
0	1 [Reference]	1 [Reference]
≥ 1	1.29 (0.95 to 1.77)	1.37 (0.96 to 1.97)
Chronic conditions linked with unemployment, No.^d^		
0	1 [Reference]	1 [Reference]
≥ 1	1.41 (1.00 to 2.00)	1.57 (1.05 to 2.34)
Cardiovascular disease		
No	1 [Reference]	1 [Reference]
Yes	1.95 (1.23 to 3.09)	2.15 (1.27 to 3.64)
Diabetes		
No	1 [Reference]	1 [Reference]
Yes	1.93 (1.15 to 3.24)	2.52 (1.43 to 4.45)
Musculoskeletal disorders^e^		
No	1 [Reference]	1 [Reference]
Yes	1.27 (0.79 to 2.03)	1.33 (0.81 to 2.20)
Hypertension		
No	1 [Reference]	1 [Reference]
Yes	0.90 (0.54 to 1.51)	1.08 (0.60 to 1.94)
Chronic respiratory disease		
No	1 [Reference]	1 [Reference]
Yes	1.43 (0.75 to 2.72)	1.11 (0.51 to 2.42)
Chronic kidney disease		
No	1 [Reference]	1 [Reference]
Yes	1.65 (0.61 to 4.50)	2.38 (0.87 to 6.54)
Neurodegenerative conditions		
No	1 [Reference]	1 [Reference]
Yes	1.81 (0.81 to 4.05)	1.59 (0.65 to 3.89)

^a^Dependent (outcome) variable is individuals who became unemployed at any time during the concurrent crises (2020–2023) compared to those who were still employed after the crises.

^b^Minimal sufficient adjustment was made to estimate the total effect of the presence and types of pre-existing chronic conditions on employment attrition during the concurrent crises; accounting for age, sex, marital status, job sector before the crises, and job income before the crises (USD per year). OR: Odds Ratio; CI: Confidence Interval.

^c^Chronic conditions (pre-existing) refer to a history of one or more of the following conditions prior to the onset of the crises: cardiovascular disease, diabetes, musculoskeletal disorders, hypertension, chronic respiratory disease, chronic kidney disease, or neurodegenerative conditions.

^d^Chronic conditions (pre-existing) linked with unemployment refer to a history of one or more of the following conditions, prior to the onset of the crises, known to be associated with early retirement or reduced labor market participation: cardiovascular disease, diabetes, or musculoskeletal disorders.

^e^A statistically significant interaction with age was identified for musculoskeletal disorders (p-value = 0.044). This overall adjusted OR does not reflect the age-dependent association. See S2 Table and Fig 3 for age-stratified odds.

**Fig 3:**
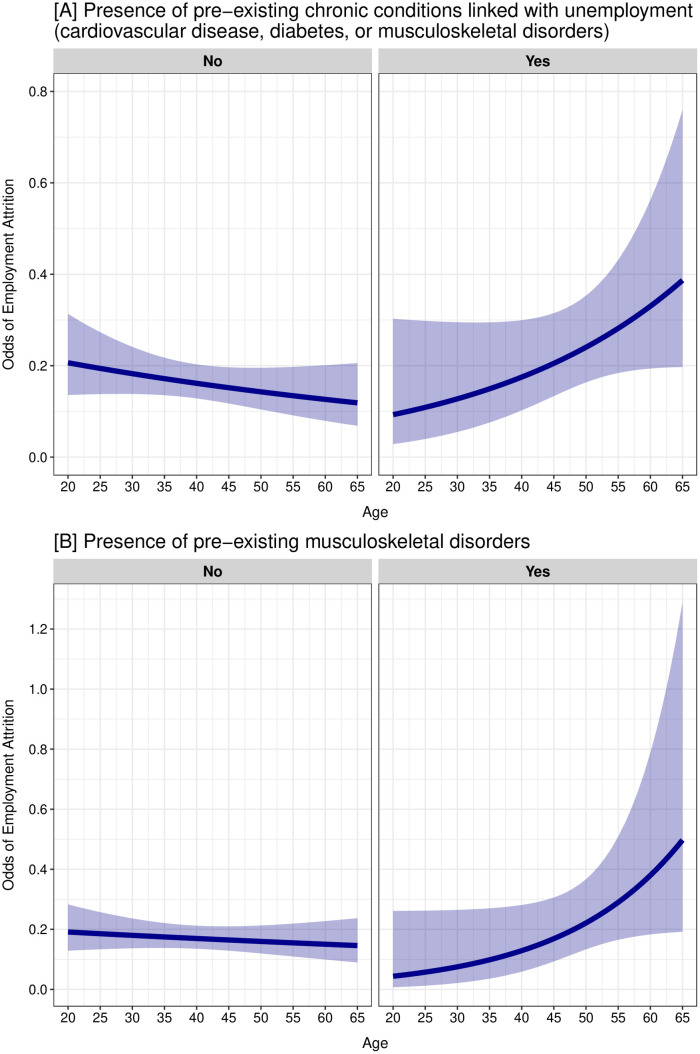
Odds of employment attrition during the concurrent crises in Lebanon (2020-2023) at different ages among individuals with and without pre-existing chronic conditions.

Participants with at least one of the broader range of pre-existing chronic conditions showed increased odds of employment attrition (aOR: 1.37; 95% CI, 0.96 to 1.97) compared to those without any of these conditions; however, these associations were not statistically significant. When considering each of these conditions individually; those with hypertension, chronic respiratory disease, chronic kidney disease, or neurodegenerative conditions had higher odds of employment attrition; however, these associations were not statistically significant. While the overall main effect for musculoskeletal disorders was not statistically significant, this estimate was modified by age, detailed in the interaction below.

There was a statistically significant interaction between age and having at least one of the pre-existing chronic conditions (CVD, diabetes, or musculoskeletal disorders) (p-value = 0.039) and having pre-existing musculoskeletal disorders (p-value = 0.044). The odds of employment attrition at different ages among individuals with and without these chronic conditions are presented in Fig 3. The ORs of employment attrition comparing those with these chronic conditions to those without were statistically significant at the 0.05 level for older ages only (S2 Table). This indicates that, at older ages, individuals with these specific chronic conditions had statistically significantly higher odds of employment attrition compared to those without. Specifically, ORs were statistically significant at ages 48 and above for having chronic conditions linked with unemployment (age 20: OR, 0.45; 95% CI, 0.13 to 1.56; age 48: OR, 1.54; 95% CI, 1.01 to 2.36; age 60: OR, 2.61; 95% CI, 1.35 to 5.07), and at ages 55 and above for having musculoskeletal disorders (age 20: OR, 0.23; 95% CI, 0.04 to 1.40; age 55: OR, 1.87; 95% CI, 1.02 to 3.44; age 60: OR, 2.52; 95% CI, 1.14 to 5.61) (S2 Table).

## Discussion

This study explored predictors of employment attrition among individuals aged 19–64 years old residing in Lebanon during the concurrent pandemic and economic crisis, between 2020 and 2023. Being of older age, female sex, non-Lebanese nationality, being married, having no formal education, having at least one of either CVD, diabetes, or musculoskeletal disorders, working in a private business or non-governmental institution, and having an oral agreement with employer were predictors of employment attrition during the crises in Lebanon. The prediction model demonstrated a moderate discriminative ability and good calibration. In addition, pre-existing CVD, diabetes, and musculoskeletal disorders were also independently associated with employment attrition.

Micro-level predictors identified in this study align with a systematic review of global evidence, which found age, gender, ethnicity, and education as potential moderators of the COVID-19 pandemic impact on individual labor market outcomes and correlates of labor force instability [17,34]. Additionally, studies examining predictors of employment outcomes have consistently shown that older age, female sex, and lower educational attainment increased the risk of unemployment [12,35]. These findings suggest that, when these vulnerabilities are already embedded at the population level, the risk of early exit from paid employment may be further exacerbated by the added complexity of crises. This was also found to be the case in Sudan and Jordan where married individuals, especially women, with children or providing care to family members, were more likely to be unemployed or leave employment during the pandemic [18,36]. These results could be explained by the increased and disproportionate care burden imposed by COVID-19 public health measures on women. In addition, this study showed meso-level factors, such as working in a private business or non-governmental institution, as predictors of employment attrition. This aligns with global and regional evidence that the pandemic and economic distress disproportionately impacted private sector workers. This sector may have been impacted disproportionally due to reduced consumer demands, as well as the higher susceptibility of private-sector workers to layoffs during economic downturns compared to the public sector [17,18].

Furthermore, having pre-existing CVD, diabetes, or musculoskeletal disorders was significantly associated with higher odds of job loss during the crises in Lebanon. Similar to Lebanon, in Egypt and Tunisia people with chronic conditions were less likely to participate in the labor force [37]. Our findings are also consistent with previous research that attributed CVD and type 2 diabetes mellitus to lower labor force participation [5,12,13]. Health conditions, especially if poorly managed, can affect employment trajectories due to disease prognosis interfering with the ability to work and the quality of life [38]. In addition, age modified the association between musculoskeletal disorders and employment attrition, suggesting that the effect of these conditions on employment attrition is stronger among older individuals. Aligned with other studies, musculoskeletal disorders such as back pain, arthritis, or joint problems were associated with earlier retirement and loss of paid employment among older workers [39,40]. These conditions increase the likelihood of functional limitations at work, rendering older individuals susceptible to job loss.

While there was an increased odds of employment attrition when exploring each type of chronic condition, the association was not statistically significant for the remaining chronic conditions – hypertension, chronic respiratory disease, chronic kidney disease, and neurodegenerative conditions. The literature is inconsistent regarding these conditions; for example, some studies showed that neurological diseases and chronic obstructive pulmonary disease predicted loss of employment or receiving disability benefits [12,41], while the association was not statistically significant in other studies [42,43]. This discrepancy could be explained by contextual differences. Furthermore, the relationship between chronic conditions and employment is intricate. Employment can secure access to healthcare through employer-sponsored insurance and disposable income for out-of-pocket spending, making employment more desirable for people with chronic conditions [44]. Conversely, having a chronic condition and its accompanying physical or mental hardship can make participation in the labor market more challenging, which further complicates this relationship. This discrepancy may also be attributed to differences in disease prognosis and how it interferes with work ability, which depend on the types of jobs involved and context-specific management strategies. For instance, in some countries such as in Germany, employees with disabilities are protected by national law against dismissal and are provided with structural support [45].

This study has its limitations. The prediction model had a moderate discriminative ability, which may be attributed to missing predictors, yet it remained comparable to prediction models of involuntary exit from paid employment applied in other contexts [12,46]. Relevant variables such as informal employment, occupational hazards, and access to healthcare should be included in future studies to improve the prediction model performance. In addition, future studies should seek to externally validate the developed model in different datasets to understand the model’s generalizability. Furthermore, this study is subject to reverse causality, despite using a DAG to delineate temporal order between pre-existing chronic conditions and employment attrition. As such, longitudinal studies are required in LMIC contexts. Additionally, the outcome of this study was self-reported, nonetheless, individuals were classified based on their job history dates to reduce information bias. The single-item self-report measure of urbanization might have introduced misclassification bias as it is based on how individuals perceive the degree of urbanization of their environment. Despite these limitations, rigorous data quality checks were conducted; 5% of the surveys were recorded and cross-checked, yielding an average error rate of only 0.6%.

## Conclusion

This nationally representative study has filled an important gap in a context where multiple crises have exacerbated inequalities in employment and health. Specifically, it has identified individuals who experience heightened vulnerability to exiting the workforce. The findings underscore the importance of addressing disparities that contribute to employment attrition and emphasize the necessity for proactive job protections to mitigate workforce disruptions during times of crises. This will advance inclusive, sustained employment and build resilience against future health threats, particularly in LMIC contexts with limited or absent social safety nets. While this cross-sectional study provides valuable insights, longitudinal studies in LMICs are necessary to investigate this relationship further.

## Supporting information

S1 Appendix Further statistical methods. (DOCX)

S1 Table Characteristics of the study sample (n = 2103). (DOCX)

S2 Table Odds ratios of employment attrition during the concurrent crises in Lebanon (2020–2023) at different ages among individuals with pre-existing^a^ chronic conditions^b^ compared to those without. (DOCX)

S1 Fig Primary reasons for leaving work during the concurrent crises among participants who experienced employment attrition during the crises (n = 265). Dismissal or redundancy refers to participants who were involuntarily terminated by their employer due to either (i) dismissal, involving conduct or capability issues such as poor performance, misconduct, or violation of company policies; or (ii) redundancy, due to company-related factors such as restructuring, downsizing, technological advancements, or decline in demands. Ending economic activity refers to participants who resigned or retired. Inappropriate working conditions includes working hours, salary, work environment, or logistical challenges accessing the workplace. Family sickness/responsibilities include participants who left their work due to family illness, caregiving, marriage, or pregnancy.
(TIF)

S2 Fig Directed Acyclic Graph (DAG) of the proposed causal model between the presence and types of chronic conditions and employment attrition during the concurrent crises in Lebanon. DAG was created using http://www.dagitty.net/. SES, socioeconomic status; Pre-2020, before the year 2020 (before the onset of the concurrent crises).
(TIF)
