# Correction: Predictors of employment attrition in Lebanon during multifaceted crises: The role of chronic diseases – a national cross-sectional study

**DOI:** 10.1371/journal.pone.0355770

**Published:** 2026-08-10

**Authors:** The PLOS One Staff

## Notice of Republication

An incorrect version of S1 and S2 File was published in error. This article was republished on July 28, 2026, to correct this error. Please download this article again to view the correct version. The publisher apologises for the error.
