# Supplementary material for: Predictors of employment attrition in Lebanon during multifaceted crises: The role of chronic diseases – a national cross-sectional study
Source: PLoS One. 2026 Mar 25;21(3):e0328028. doi: 10.1371/journal.pone.0328028 (PMC13016281; doi:10.1371/journal.pone.0328028)
Supplement: S1 Appendix — (DOCX) [file pone.0328028.s001.docx]

**Materials and methods**

**S1 Appendix. LASSO Methodological Details.**

The penalty strength was selected using 10-fold cross-validation. The cross-validation evaluated different penalty strength (λ) values to identify the optimal value that minimized the model’s misclassification error (optimal λ = 0.0003507). The LASSO logistic regression retained variables and handled multicollinearity by shrinking the coefficients of correlated predictor variables towards zero. Model convergence was assessed by monitoring the stability of coefficients across iterations to ensure that the optimized algorithm reached stable values^[[1]](#footnote-1)^.

The final model performance was assessed through its discrimination and calibration abilities^[[2]](#footnote-2)^. A model’s discriminative ability was assessed using the C-Statistic/Area Under the Receiver Operating Characteristic Curve (AUC), with a value of 1 indicating perfect discrimination ability. Calibration was assessed by comparing predicted and observed values; a perfectly calibrated model follows a diagonal line with an intercept of 0 and a slope of 1. A slope less than 1 suggests model overfitting (overestimated predictions for high-risk individuals and underestimated predictions for low-risk individuals), and the opposite for slopes higher than 1. Calibration-in-the-large (CITL) was used to assess whether the overall number of observed outcome events matched the predicted risk.

1. Tibshirani R. Regression shrinkage and selection via the lasso. Journal of the Royal Statistical Society Series B: Statistical Methodology. 1996 Jan;58(1):267-88. [↑](#footnote-ref-1)
2. Steyerberg EW, Vickers AJ, Cook NR, Gerds T, Gonen M, Obuchowski N, et al. Assessing the performance of prediction models: a framework for traditional and novel measures. Epidemiology. 2010;21(1):128-38. [↑](#footnote-ref-2)
