# Supplementary material for: Predictors of employment attrition in Lebanon during multifaceted crises: The role of chronic diseases – a national cross-sectional study
Source: PLoS One. 2026 Mar 25;21(3):e0328028. doi: 10.1371/journal.pone.0328028 (PMC13016281; doi:10.1371/journal.pone.0328028)
Supplement: S1 Table — (DOCX) [file pone.0328028.s002.docx]

**S1 Table. Characteristics of the Study Sample (n = 2103).**

|  | | **Participants, No. (Weighted %)** |
| --- | --- | --- |
|  |  | **Total (n = 2103)** |
| Age, median (IQR), years | | 40 (33-49) |
| Sex | |  |
| Male | | 1466 (72.7) |
| Female | | 637 (27.3) |
| Nationality | |  |
| Lebanese | | 1311 (70.1) |
| Non-Lebanese | | 792 (29.9) |
| Marital status | |  |
| Married | | 1588 (75.2) |
| Non-married^a^ | | 515 (24.8) |
| Education | |  |
| Never attended school | | 67 (2.9) |
| Formal or technical education | | 1500 (70.9) |
| Higher education | | 536 (26.2) |
| Urbanization of living environment | |  |
| Not urbanized/strongly urbanized | | 1642 (80.0) |
| Extremely urbanized | | 439 (20.0) |
| Missing | | 22 |
| Children, median (IQR), No. | | 2 (1-4) |
| Pre-existing^b^ chronic conditions linked with unemployment, No.^c^ | |  |
| 0 | | 1786 (84.6) |
| ≥ 1 | | 310 (15.4) |
| Missing | | 7 |
| Cardiovascular disease, pre-existing | |  |
| No | | 1961 (93.4) |
| Yes | | 125 (6.6) |
| Missing | | 17 |
| Diabetes, pre-existing | |  |
| No | | 1995 (94.9) |
| Yes | | 94 (5.1) |
| Missing | | 14 |
| Musculoskeletal disorders, pre-existing |  | |
| No | | 1924 (92.1) |
| Yes | | 167 (7.9) |
| Missing | | 12 |
| Job sector, pre-2020^b^ | |  |
| Government | | 271 (14.1) |
| Private business | | 978 (46.6) |
| Private household | | 60 (2.4) |
| Non-governmental institution | | 75 (3.6) |
| Freelance | | 715 (33.3) |
| Missing | | 4 |
| Contractual agreement, pre-2020 | |  |
| Written | | 489 (25.1) |
| Oral | | 921 (42.4) |
| Independent contractors^d^ | | 651 (32.5) |
| Missing | | 42 |
| Employment attrition during the crises | |  |
| Remained employed after the crises | | 1838 (85.3) |
| Left employment during the crises | | 265 (14.7) |

^a^ Non-married includes participants who are single, engaged, divorced/separated, or widowed.

^b^ Pre-existing or pre-2020 refers to conditions or circumstances that were present prior to the onset of the concurrent crises, before the year 2020.

^c^ At least one of the chronic conditions known to be associated with early retirement or reduced labor market participation: cardiovascular disease, diabetes, or musculoskeletal disorders.

^d^ Independent contractors include owners, partners, and own account workers.
