# Supplementary material for: Predictors of employment attrition in Lebanon during multifaceted crises: The role of chronic diseases – a national cross-sectional study
Source: PLoS One. 2026 Mar 25;21(3):e0328028. doi: 10.1371/journal.pone.0328028 (PMC13016281; doi:10.1371/journal.pone.0328028)
Supplement: S2 Table — (DOCX) [file pone.0328028.s003.docx]

**S2 Table. Odds Ratios of Employment Attrition During the Concurrent Crises in Lebanon (2020-2023) at Different Ages Among Individuals with Pre-existing^a^ Chronic Conditions^b^ Compared to Those Without.**

|  | **Age (years)** | **OR (95% CI)** | **p-value** |
| --- | --- | --- | --- |
| Having at least one chronic condition linked with unemployment (cardiovascular disease, diabetes, or musculoskeletal disorders) | 20 | 0.45 (0.13 to 1.56) | 0.207 |
|  | 25 | 0.56 (0.20 to 1.60) | 0.279 |
|  | 30 | 0.70 (0.30 to 1.65) | 0.413 |
|  | 35 | 0.87 (0.44 to 1.73) | 0.692 |
|  | 40 | 1.08 (0.63 to 1.86) | 0.767 |
|  | 45 | 1.35 (0.87 to 2.10) | 0.182 |
|  | 50 | 1.68 (1.09 to 2.60) | 0.019* |
|  | 55 | 2.10 (1.25 to 3.53) | 0.005* |
|  | 60 | 2.61 (1.35 to 5.07) | 0.004* |
|  | 64 | 3.12 (1.40 to 6.92) | 0.005* |
| Having musculoskeletal disorders | 20 | 0.23 (0.04 to 1.40) | 0.111 |
|  | 25 | 0.31 (0.07 to 1.44) | 0.134 |
|  | 30 | 0.42 (0.12 to 1.48) | 0.176 |
|  | 35 | 0.56 (0.21 to 1.54) | 0.264 |
|  | 40 | 0.76 (0.35 to 1.65) | 0.488 |
|  | 45 | 1.03 (0.57 to 1.86) | 0.930 |
|  | 50 | 1.39 (0.82 to 2.34) | 0.223 |
|  | 55 | 1.87 (1.02 to 3.44) | 0.044* |
|  | 60 | 2.52 (1.14 to 5.61) | 0.023* |
|  | 64 | 3.21 (1.20 to 8.60) | 0.021* |

^a^ Pre-existing refers to conditions that were present before the onset of the concurrent crises, prior to the year 2020.

^b^ We tested for an interaction between the presence and types of chronic conditions and age. For significant interactions (p-value < 0.05), we estimated the ORs of employment attrition comparing those with to those without the exposures at selected ages between 19 and 64 years.
OR: Odds Ratio; CI: Confidence Interval.

*Significant at the 0.05 level.
