# Supplementary figures and images for: Predictors of employment attrition in Lebanon during multifaceted crises: The role of chronic diseases – a national cross-sectional study
Source: PLoS One. 2026 Mar 25;21(3):e0328028. doi: 10.1371/journal.pone.0328028 (PMC13016281; doi:10.1371/journal.pone.0328028)

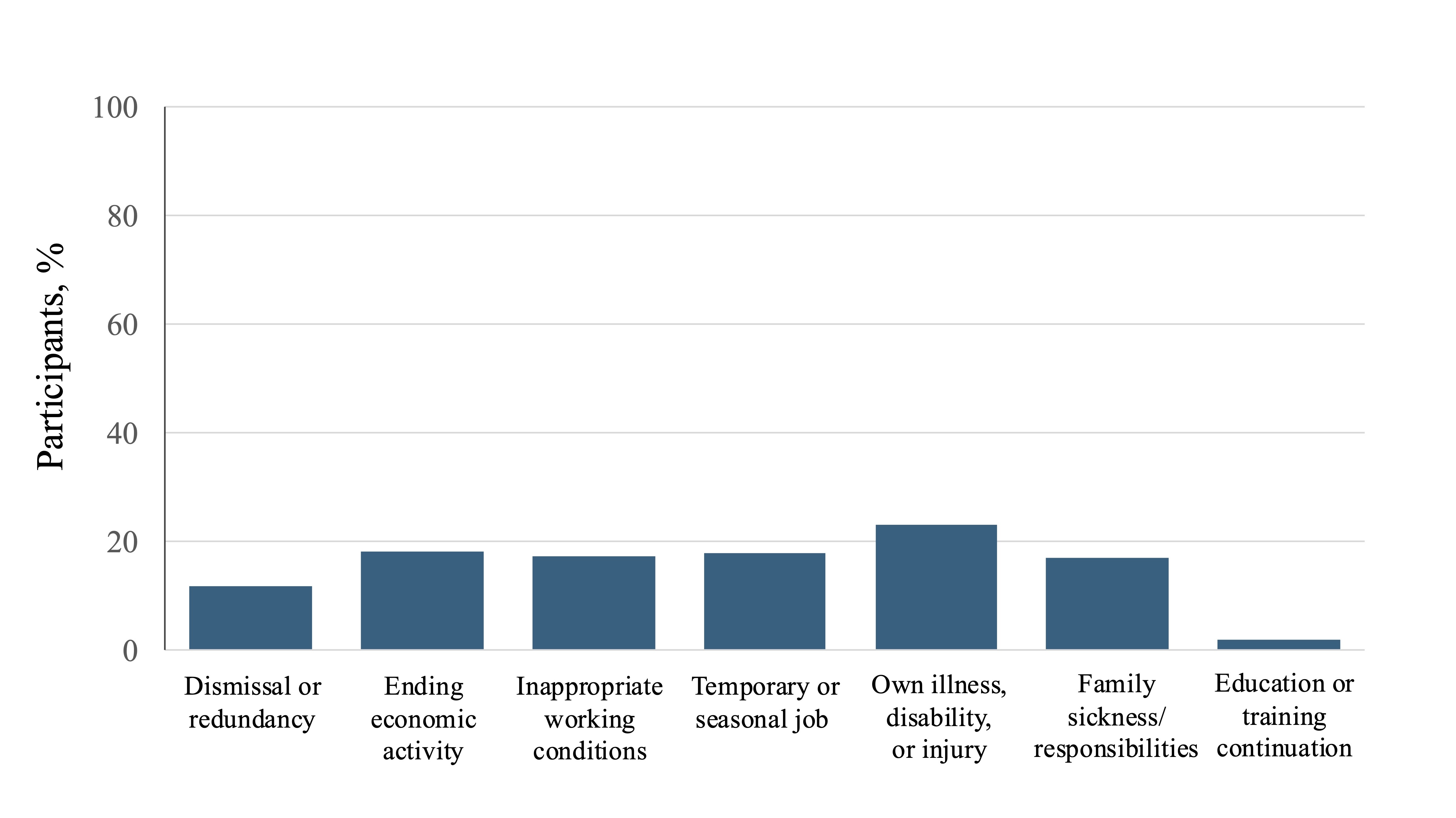

Supplement: S1 Fig — Dismissal or redundancy refers to participants who were involuntarily terminated by their employer due to either (i) dismissal, involving conduct or capability issues such as poor performance, misconduct, or violation of company policies; or (ii) redundancy, due to company-related factors such as restructuring, downsizing, technological advancements, or decline in demands. Ending economic activity refers to participants who resigned or retired. Inappropriate working conditions includes working hours, salary, work environment, or logistical challenges accessing the workplace. Family sickness/responsibilities include participants who left their work due to family illness, caregiving, marriage, or pregnancy. (TIF) [file pone.0328028.s004.tif]

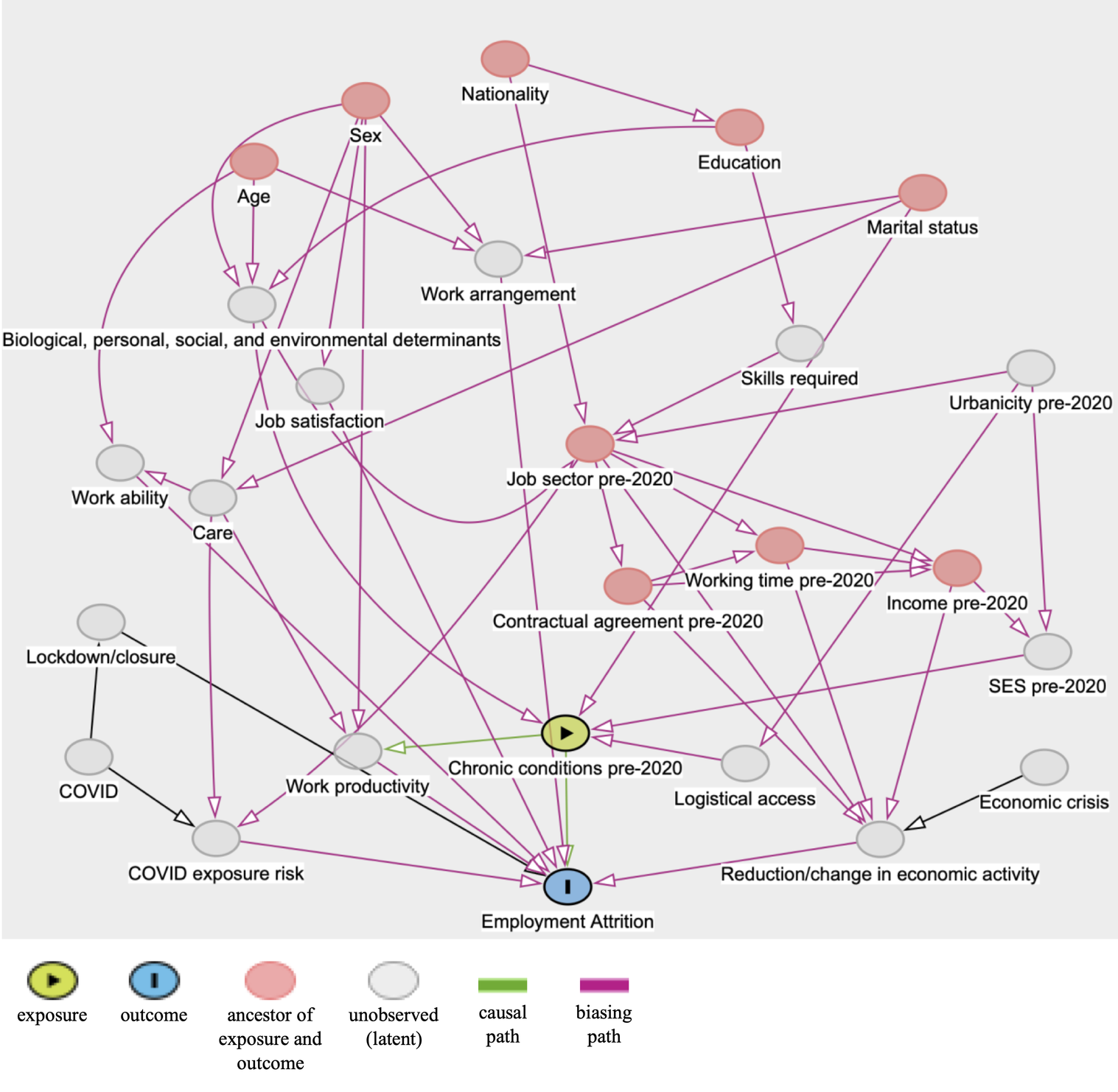

Supplement: S2 Fig — DAG was created using http://www.dagitty.net/. SES, socioeconomic status; Pre-2020, before the year 2020 (before the onset of the concurrent crises). (TIF) [file pone.0328028.s005.tif]
